# Supplementary material for: Prolonged recovery time after eruptive disturbance of a deep-sea hydrothermal vent community
Source: Proc Biol Sci. 2020 Dec 23;287(1941):20202070. doi: 10.1098/rspb.2020.2070 (PMC7779506; doi:10.1098/rspb.2020.2070)
Supplement: Supplementary Data Tables 1-4 [file rspb20202070supp1.docx]

Mullineaux, L.S., S.W. Mills, N. Le Bris, S.E. Beaulieu, S.M. Sievert, and L.N. Dykman, 2020

Prolonged recovery time after eruptive disturbance of a deep-sea hydrothermal vent community

Proceedings of the Royal Society B

DOI 10.1098/rspb.2020.2070

Supplementary Data Tables 1-4

Supplementary Data Table 1. Abundance of species and morphogroups on post-eruption sandwiches (at P-vent) and pre-eruption blocks (at East Wall). Recovery date (months since 2006 eruption), deployment habitat (H=Hot, W=Warm, C=Cool), sample ID, and recovery temperature (°C) are presented for each sample. ND = no temperature data recorded

*may include small *Protis hydrothermica*

Supplementary Data Table 2. Kruskal Wallace (K-W) analysis of variation in abundance over time of prominent species colonizing sandwiches at P-vent.

^*^Values selected from habitat zone where each species occurred most commonly (Hot for *Ctenopelta porifera*, Warm for all others).

^**^Bolded when significant at P < 0.05 level; Df = 4 for *Ctenopelta porifera*; Df = 5 for all othersSupplementary Data Table 3. Pearson correlation matrices for nMDS plots from Hot (a), Warm (b) and Cool (c) habitat, corresponding to Fig. 4 a-c. Samples designated by date (months post-eruption, or pre-eruption) and recovery temperature (°C).

a) Hot

b) Warm

c) Cool

Supplementary Data Table 4. Species from regional pool near P-vent that were not observed as colonists during the initial 11 years after the 2006 eruption. List was filtered from Desbruyères et al (2006) to include only species identifiable by the techniques used in this study and in the size range (macrofauna and larger), mobility category (sedentary), and thermal environment (<30℃) sampled by sandwiches. Region included 9°N and 13°N. A total of 91 species and morphogroups, including the 68 found as colonists in pre- and post-eruption monitoring, are included in the relevant species pool.

| **Species** |
| --- |
| *Neolepetopsis densata* McLean, 1990 |
| *Lepetodrilus galriftensis* McLean, 1988 |
| *Melanodrymia galeronae* Warén & Bouchet, 1993 |
| *Neomphalus fretterae* McLean, 1981 |
| *Echinopelta fistulosa* McLean, 1989 |
| *Hirtopelta hirta* McLean, 1989 |
| *Lirapex granularis* Warén & Bouchet, 1989 |
| *Nodopelta heminoda* McLean, 1989 |
| *Nodopelta rigneae* Warén & Bouchet, 2001 |
| *Nodopelta subnoda* McLean, 1989 |
| *Peltospira lamellifera* Warén & Bouchet, 1989 |
| *Peltospira operculata* McLean, 1989 |
| *Calyptogena magnifica* Boss & Turner, 1980 |
| *Eunice pulvinopalpata* Fauchauld, 1982 |
| *Amphiduropsis axialensis* Blake & Hilbig, 1990 |
| *Levensteiniella plicata* Hourdez & Desbruyères, 2000 |
| *Oasisia alvinae* Jones, 1985 |
| *Dahlella caldariensis* Hessler, 1984 |
| **Munidopsis subsquamosa* Henderson, 1885 |
| *Bythograea microps* De Saint Laurent, 1989 |
| *Cyanagraea praedator* De Saint Laurent, 1984 |
| *Freyella* Perrier, 1885 |
| *Saxipendium coronatum* Woodwick & Sensenbaugh, 1985 |
|  |
| * listed as *Munidopsis* in Desbruyères et al. 2006 |
